# Impact of Equine-Assisted Interventions on Heart Rate Variability in Two Participants with 22q11.2 Deletion Syndrome: A Pilot Study

**DOI:** 10.3390/children8111073

**Published:** 2021-11-22

**Authors:** Maria Amado-Fuentes, Margarita Gozalo, Andres Garcia-Gomez, Sabina Barrios-Fernandez

**Affiliations:** 1 Psychology and Anthropology Department, University of Extremadura, 10003 Cáceres, Spain; mamadofu@alumnos.unex.es; 2 Occupational Stress, Psychopathologies and Emotional Well-Being (GRESPE) Research Group, University of Extremadura, 06006 Badajoz, Spain; 3 Social Impact and Innovation in Health (InHEALTH) Research Group, University of Extremadura, 10003 Cáceres, Spain; sabinabarrios@unex.es

**Keywords:** equine-assisted interventions, stress, heart rate variability, rare diseases, 22q11.2 deletion syndrome

## Abstract

People with disabilities due to genetic origin often present high levels of stress: non-pharmacological interventions such as Equine-Assisted Interventions (EAI) may be a useful strategy. The objective of this pilot study was to evaluate stress levels in two participants with 22q11.2 deletion syndrome diagnosis, immediately after carrying out the EAI. A single case experimental design methodology was chosen due to the small sample size. Two participants with 22q11.2 Deletion Syndrome, a rare disease, with different comorbidities were included. The present study considered the EAI as the independent variable while the Heart Rate Variability (HRV) represented the dependent one, as HRV is considered an indicator of stress level. Measurements were performed before and after carrying out the interventions. The results showed an HRV increase in one of the participants and an increase in the arousal level evidenced by a decrease in his HRV. After having carried out the program, EAI seems to cause an impact on the activation level of the participants depending on the typology and nature of the intervention. However, these results should be treated with caution due to the small sample size. This study is a pilot to test the feasibility of the proposed interventions on the variable under study.

## 1. Introduction

Animal-assisted interventions, including horses, are becoming increasingly popular in our society and there is a growing number of centers and users practicing them. This growth has occurred not only at the clinical level but also in research [1,2]. Equine-assisted interventions (EAI) have focused on three areas: equine-assisted therapies, equine-assisted education and learning, and adapted horseback riding [3]. EAIs are showing promising results in physical and psychological conditions [4], benefits being evidenced in pathologies’ core symptoms as well as in symptomatology related to adaptation difficulties [5,6,7,8,9,10,11].

People with genetic rare diseases have also been studied in the context of animal-assisted interventions. Some research has shown that both interactions with animals and adapted horseback riding practice have beneficial effects on the health and quality of life of people who are part of this heterogeneous group [12,13,14,15]. However, many challenges remain.

People with 22q11.2 Deletion Syndrome (22q11.2 DS) are part of the group of people with rare diseases. This syndrome, also known as DiGeorge and velocardiofacial syndrome, is characterized by the deletion or loss of genetic material on chromosome 22, specifically in a region called 22q11.2. In this region, there are about 50 genes involved in various aspects of embryonic development. 22q11.2DS is one of the most common microdeletions in humans, with a prevalence ranging from 1/2000–4000 live births and around 1/1000 pregnancies [16]. Although this syndrome presents a great phenotypic heterogeneity, individuals with this syndrome usually present similar facial features, such as elongated face, expressionless face, prominent ears, or broad nasal bridge, although they are not always present. Common clinical manifestations include heart disease, palate abnormalities, immunodeficiency, hypocalcemia, and other neuropsychiatric and cognitive disorders such as Parkinson’s disease, intellectual disability, epilepsy, autism spectrum disorder (ASD), attention deficit hyperactivity disorder (ADHD), coordination disorders, speech and language disorders, anxiety, and schizophreniform disorder [17,18,19,20,21].

As in other disabilities, people with 22q11.2 DS often present high levels of stress and anxiety. Adaptation difficulties presented by people with disabilities are powerful stressors in daily life, which often results in suffering from chronic situations of anxiety in some cases inseparable from the disorder [22,23,24,25,26]. In addition, anxiety must be understood systemically, as it is related to the severity of the symptoms and affects not only the individual but also the whole family [27,28,29,30]. Stress and anxiety study need to be considered as they decisively affect the course of the disorder [31], and it is related to a decrease in cognitive and social skills [32,33,34], in addition to being a triggering factor for comorbidity [22,35,36,37].

Stress and anxiety in the 22q11.2 DS population have been managed with pharmacological treatments [38], cognitive-behavioural treatments [39], through specialized educational resources, and social support [40]. Both pharmacological and non-pharmacological alternatives are still needed for this population, which is generally heavily medicated due to the high concurrence of comorbidities [41,42].

Recent work has attempted to assess the impact of EAI on individuals’ stress with promising preliminary results. These studies have assessed participant stress through observational questionnaires and objective biological variables such as cortisol [11,43,44,45] and Heart Rate Variation (HRV) [15]. Moreover, we have not found studies on the EAIs’ impact on stress levels in subjects with 22q11.2 DS diagnosis. Therefore, the present study aims to assess the EAIs’ impact on HRV in the two participants with 22q11.2 DS and therefore on their stress levels, implementing a pilot study with a small sample to check the feasibility of a larger study and provide preliminary data on an unexplored population.

## 2. Materials and Methods

### 2.1. Design

A single case study with two 22q11.2 DS diagnosed participants was chosen to assess EAI impact on participants’ HRV as an indicator of stress. Different parameters of HRV were assessed before and after the interventions. Therefore, the EAI program was taken as the independent variable while HRV was the dependent one.

### 2.2. Variables

To facilitate interpretation of the variables, it is useful to clarify that Heart Rate (HR) is the one at which the heart beats during a certain period of time. Since the interval between beats is not always the same, the Heath Rate Variation (HRV) refers to the variation in these time intervals between each beat. The most common way to measure this variability is with an electrocardiogram (ECG), which detects each of the N-waves (the voltage expressed in each beat) and calculates the time between each consecutive N-wave, (i.e., the NN intervals) [46]. Detailed analysis of HRV allows studying the activity of the Autonomic Nervous System (ANS). ANS activity is based on the balance between the sympathetic nervous system (SNS) and the parasympathetic nervous system (PNS). In a resting state, stimulation of the PNS predominates, whereas in a state of anxiety, stress or physical exercise, stimulation of the SNS predominates [47]. Low HRV conveys a regular and monotonous heartbeat that is associated with alterations in regulatory functions and the ANS, which reduces the system’s ability to cope with internal and external stressors. On the other hand, a high HRV is associated with activation of the PNS, i.e., a state of both physical and psychological relaxation [48]. The best indicators of HRV and the most reported in preceding studies are (1) time domain-based parameters, and (2) frequency domain-based parameters [15]. Time domain-based parameters include the standard deviation of all interbeat intervals (SDNN), the square root of the mean of the sum of the squares of the differences between adjacent intervals (RMSSD). The higher the score on these parameters, the higher the HRV.

The parameters in the frequency domain are based on the decomposition of the energy (power) of the NN signal in different frequency components: the power of the low-frequency range (range 0.04–0.15 Hz) in ms^2^ (LF), the power of the high-frequency range (range 0.15–0.4 Hz) in ms^2^ (HF), and the LF/HF ratio. LF has been related to the activation of the SNS and of the PNS. HF exclusively reflects the contribution of the PNS, and LF/HF ratio measures the participation of the sympathetic and parasympathetic nervous system, with the understanding that the higher the ratio (greater than 1), the more the SNS is controlled. In addition to these variables, we offer the Baevsky and Berseneva stress index [49], The Baevsky and Berseneva stress index (1) is based on a series of geometric means that are calculated from the histogram of the interbeats interval (NN). The following formula is used to estimate this stress index [49,50,51], where a higher score on the stress index corresponds to a higher level of stress.

(1) $$\mathrm{SI}=\frac{\mathrm{Amo}\times 100\%}{2\mathrm{Mo}\times \mathrm{MxDMn}}$$

where:

- Amo: width of the most frequent NN interval in percentage terms.
- Mo: most frequent NN interval.
- MxDMn: difference between the values of the longest and the shortest NN interval.

### 2.3. Participants

The participants were selected from among the users of a riding centre using a non-probability sampling method based on convenience sampling based on the following eligibility criteria: (a) having 22q11.2 Deletion Syndrome diagnosis, (b) being able to follow simple commands, (c) motor autonomy to perform EAI on foot or horseback, (d) absence of phobias, allergy, or other conditions incompatible with interaction with horses, (e) personal motivation to participate, (f) providing informed consent signed by parents or legal guardians.

The clinical diagnosis of both users was initially performed by fluorescence in situ hybridisation (FISH) and later confirmed with NEAUROARRAY^®^ cytogenetic-molecular diagnosis. The samples were analysed in the Genetadi Biotech S.L. laboratories (Derio, Vizcaya, Spain), using the Array Comparative Genome Hybridization (aCGH 400K) technique with the Agilent CytoGenomics software (Agilent Technologies, Santa Clara, CA, USA). Copy number variants (CNVs) outside the 22q11.2 region were compared to CNVs in the human genome (http://projects.tcag.ca/variation/ (accessed on 17 November 2021)). Microarray confirmed a typical deletion without duplications in both participants. Parental genetic studies indicated de novo deletions in both cases.

Finally, two participants meet the eligibility criteria. Their characteristics are presented in Table 1.

### 2.4. Instruments

Due to the cognitive difficulties of participants, a portable, reliable, and non-invasive instrument for the objective measurement of stress was chosen. The CorSense^®^ is a sensor created by Elite HRV (Gloucester, MA, USA) which takes measurements and send them to the Elite HRV application via Bluetooth (Gloucester, MA, USA). Then, the information was exported to the Kubios HRV Standard v 3.5 computer program (Kuopio, Eastern Finland). CorSense^®^ records HRV by photoplethysmography, a technique that provides information on HRV when a green light is projected on the user’s skin [52]. Kubios HRV is a scientifically validated application for HRV analysis widely used by researchers around the world. The software has been developed over the last 20 years and is used in more than 1200 universities in 128 countries [46,53,54,55,56]. Using data obtained by CorSense and analyzed by Kubios is possible to study the main parameters associated with HRV proposed by the European Society of Cardiology and the American Society of Pacing and Electrophysiology working group [49].

### 2.5. Procedure

According to the Helsinki Declaration of 2013 [57], the parents signed and delivered the informed consent form before starting the sessions. This project has the approval of the Bioethics and Biosafety Commission of the University of Extremadura with registration number 165/2020.

The data collection process lasted three months, from October to December 2020, with several breaks due to COVID-19; six samples were collected in each of the participants, although only five were recorded in one of the participants due to his absence in one session. The sessions were individual, with a duration of 45 min. HRV was measured in two moments: before and after the sessions. Each participant received a different treatment led by a special education teacher and a riding instructor with complementary training in equine-assisted interventions.

Participant 1 received the EAIs on foot only. The sessions followed this scheme: (1) collecting HRV data, (2) handling the horse and brushing and grooming the horse, (3) performing interaction activities with the instructor, (4) performing circuits guiding the horse on the ground with the short lead rope, (5) unharnessing and farewell, (6) collecting HRV data at the end of the session. All activities are carried out while maintaining communicative interactions with the support of pictograms and signs.

Participant 2 performed activities on foot and horseback. The sessions followed this scheme: (1) collecting HRV data, (2) handling the horse, brushing, grooming, and tacking of the horse (riding activities were performed with blanket and surcingle), (3) performing warm-up activities on the horse,(4) performing balance activities on the horse, (5) learning skills related to autonomy on the horse and games with horses, (6) unharnessing and farewell, (7) collecting HRV data at the end of the session.

### 2.6. Data Analysis

The data were analyzed using the software Kubios. We selected those variables most frequent in HRV studies with EAIs [52]: heart rate (HR), time domain-based parameters SDNN, RMSSD, frequency domain-based parameters LF, HF, and LF/HF ratio, and Baevsky and Berseneva stress index.

The Statistical Package for the Social Sciences (SPSS, Version 25, IBM SPSS, Armonk, NY, USA) software was used. Data are presented in graphs. The Wilcoxon test was performed to test paired samples.

As probability of significance (p) is an unreliable statistic in single-case studies since it is related to the sample size, the magnitude of the effect is the statistic of choice in this type of study. Cohen’s *d* was carried out to reflect the magnitude of the effect [58], using interpretation for single-case studies [59]: small 0–0.99; medium 1–2.49 and large <2.50.

## 3. Results

### 3.1. Participant 1

In this participant, as shown in Table 2, there are no appreciable changes in HR after the intervention sessions with horses (HR: *d* = 0.172). However, a significant and large effect was observed after the horse intervention, evidenced by the increase in several parameters of HRV, which can be related to parasympathetic nervous system activation (SDNN: *d* = 2.795, *p* = 0.046; RMSSD: *d* = 2.795, *p* = 0.046; HF: *d* = 2.795, *p* = 0.046) and by the decrease in stress levels (*d* = 2.795, *p* = 0.046).

**Figure 1:**
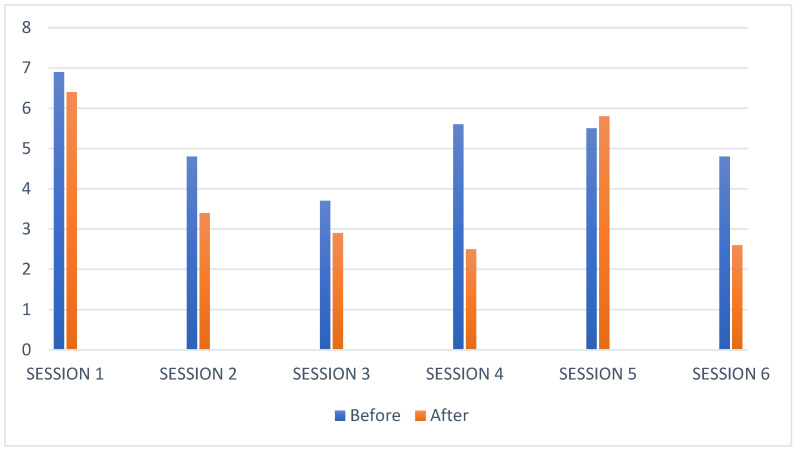
Participant 1 stress level bar chart.

Figure 1 shows that in five of the six sessions, stress levels were significantly lower after the EAI sessions.

### 3.2. Participant 2

This participant showed a slight increase in HR after the intervention, but not significant (*d* = 0.368, *p* = 0.686), as shown in Table 3. However, a moderate decrease in HRV main indicators related to parasympathetic nervous system activation was observed (SDNN: *d* = 1.293, *p* = 0.225; RMSSD: *d* = 1.293, *p* = 0.225; HF: *d* = 0.931, *p* = 0.345). This decrease in HRV values was reflected in the stress index, which showed a moderate increase after performing the EAI (*d* = 1.772, *p* = 0.138).

**Figure 2:**
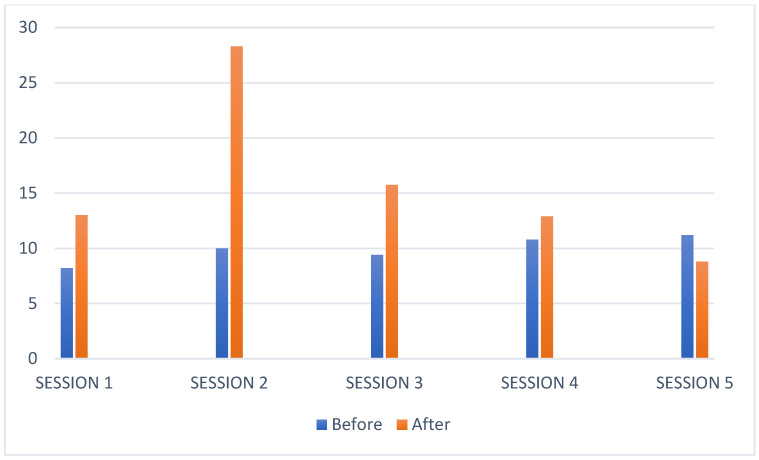
Participant 2 stress level bar chart.

As shown in Figure 2, the stress index was higher after EAI in four of the five sessions recorded.

## 4. Discussion

The present study aimed to evaluate the impact of EAI on HRV and stress levels in two participants with DS 22q11.2. To assess stress levels, the Baevsky and Berseneva stress index was used [49]. The results showed that in participant 1, HRV increases immediately after the EAI sessions and consequently their stress levels decrease moderately. However, in participant 2, there is a slight decrease in HRV and a slight increase in stress levels. The increase in HRV after EAI in participant 1 is in line with the results reported in previous research [60,61,62,63,64,65,66]. The intervention modalities developed in these studies have been based on interactive activities with horses on foot or with passive hippotherapy modalities, that is, without voluntary control of the horse by the participant but assisted by a guide who directs and controls the horse’s movements. These studies highlight that EAI increases PNS activation and therefore, a stress decrease a decrease in stress could be inferred. This effect could be favored by affective interaction with animals in a natural setting, with friendly and predictable interactions and in a highly structured environment [15]. The PNS activation must be considered since this system is responsible for restoring balance in the organism in stressful situations and, at the same time, helps to reach cognitive and emotional regulation [67].

This effect of decreased HRV has been extensively studied in sport riding activities [68,69,70,71,72]. However, in previous studies in which HRV was measured immediately after therapeutic riding activities with active handling of the horse by the participant, the results were either inconclusive or showed slight HRV decreases and thus higher levels of activation [73,74].

The activation level in participant 2 after horseback riding could be related to the eustress (positive stress) that happens immediately after the practice of physical activity. This eustress has its basis in a physiological concept called cardiac coherence, different from the relaxation state, which generally involves the active engagement of positive emotions [75]. It should be noted that especially in participant 2, the results may have been influenced by circadian changes in HRV induced by Parkinsonism and by the effect of regularly administered medication [76].

When stress is assessed by HRV at a distance from physical activity, around 60 min of inactive recovery after exercise [77], or by the cumulative effect in the medium and long term (more than one month), a significant effect of active therapeutic riding on baseline HRV levels has been demonstrated [78]. Continued exercise achieves an increase in parasympathetic activity, which exerts a protective effect on health problems and improves the quality of life of patients [79,80]. Our results revealed that, in the case of structured interactions with horses, without the involvement of physical exercise (participant 1), a decrease in stress should be observed (significant increase in HRV). However, when moderate active physical exercise happens, results indicate a slight level of activation reflected by HRV decrease (participant 2). Therefore, when performing EAIs, it is important to control the type of physical activity performed [74] since this produces a eustress situation that favors motivation, task involvement, and attention, skills that can be considered to work on executive deficits [81].

Furthermore, some authors have pointed out that EAI can be considered an alternative therapeutic tool, thanks to the patient-horse-instructor relationship and the sensory-motor and cognitive stimulation that reinforces learning processes [45], as the population with disabilities requires complementary and alternative interventions due to their high consumption of drugs [82]. Given the current state of research, it would be of interest to study if EAI may be an effective intervention that could be considered along with current standard therapies, which implies a research effort in this area.

This research has some limitations. The small sample size prevents the results to be generalized: they should only be taken as a trend to formulate new hypotheses due to the pilot study nature of this work. The study design was limited to data collection, and there was no control group. The data were taken before and after de EAIs, not assessing participants’ stress levels at different periods after the intervention. Moreover, the participants’ characteristics may also have influenced the results. Furthermore, to address the above-mentioned limitations, and once a pilot study has been carried out to check the feasibility of the project, the sample should be increased, performing an experimental design with a control group and alternative treatments within EAIs (without riding, riding, with different levels of physical activity) or comparing them with other recreational or sports activities. The research design together with the recording device should offer the possibility of recording data at different time points (before, during, immediately after the sessions, and at different times after the sessions). Moreover, it may be of great interest to study the chronic effect of the intervention on the baseline stress levels of the participants in a time interval greater than one month.

## 5. Conclusions

EAI produced an observable effect on participants’ HRV as a measure of stress level, immediately after the performance of the activities. Due to the design of the study, these data should be taken as preliminary since the conditions under which the research was carried out do not allow the generalization of the results. However, their behaviours have shown some specificity depending on the characteristics of each participant and the activity that was being developed.

Concerning each participant:

- Participant 1, who performed the EAIs without riding, showed a great magnitude of effect, with an increase in HRV before the session and lower stress levels after the EAI intervention.
- Participant 2, who rode horses, showed a moderate size of effect after the EAI on HRV and, therefore, on the level of stress, with a higher level of activation evidenced by a decrease in HRV.

A procedural conclusion should be that interactions with horses, either when the user interacts with the horse on the ground or when handling or riding the horse, have an influence on the participants’ HRV, and this influence can be objectified by non-invasive recording procedures that can be used in natural contexts.

## Figures and Tables

**Table 1:** Sociodemographic and clinical characteristics of participants.

	Participant 1	Participant 2
Age (years)	9	20
Gender	Male	Male
Diagnosis	22q11.2 DS	22q11.2 DS
Comorbidities	Autism Spectrum Disorder, Grade 2 Moderate Intellectual Disability	Parkinsonism. Partial paralysis of a vocal cord and problems in glottic closure. Obsessive Compulsive Disorder. Depression with psychotic symptoms. Mild Intellectual Disability.
Medication	Casenlax^®^ 10 g (polyethylene glycol).	Sinemet^®^ (carbidopa-levodopa), (break/)Nemea^®^ (Clozapine), Fluoxetine.
Gross motor development	Generalized hypotonia. Walking difficulties (uses Dynamic Ankle Foot Orthosis). Coordination issues.	Walks with flexed head and without an arm swing. High dorsal kyphosis and compensatory lumbar lordosis. Generalized bradykinesia. Coordination and clumsiness issues.
Fine motor development	Poor dexterity and bimanual coordination	Poor dexterity and bimanual coordination
Social and adaptive development	Relationships with adults, peer interactions should be encouraged and supported. Substantial support in daily life activities.	A narrow range of friendships. Poor social skills. Sometimes shares his interests. Lack of initiative and anticipation. Needs occasional support in daily life activities.
Language and communication development	Uses oral language with holophrases, pictograms and signs support.	Low communicative intentionality. Problems in the breath/phonation coordination. Issues sharing emotions.
Sensory integration	Searching behaviors. Hyperreactivity.	Hyperreactivity.
Schooling modality	Regular school with therapeutic pedagogy and speech therapy teachers’ assistance. Individualized curricular adaptation	Training in administration and finance.

**Table 2:** Participant 1 statistics and contrast for HRV-related parameters.

Parameters	Descriptives		Wilcoxon’s Test		
	Before Intervention	After Intervention	*Z*	*p*	Cohen’s d
HR (bpm)	89.16 ± 4.53	89.33 ± 7.11	−0.21	0.833	0.172
SDNN (ms)	133.83 ± 19.13	215.25 ± 71.90	−1.992	0.046	2.795
RMSSD (ms)	170.08 ± 16.52	248.81 ± 68.41	−1.992	0.046	2.795
LF (ms^2^)	6954.16 ± 5966.5	49,849.5 ± 52,631.53	−1.572	0.116	1.674
HF (ms^2^)	6482.83 ± 3676.8	121,090.33 ± 148,075.62	−1.992	0.046	2.795
LF/HF (ms^2^)	0.999 ± 0.57	1.17 ± 1.21	−0.105	0.917	0.086
Stress	5.2 ± 1.0	3.93 ± 1.71	−1.992	0.046	2.795

*p* < 0.05 Cohen’s *d*, considering: small 0–0.99, medium 1–2.49, and large +2.50 [59]. HR: heart rate (bpm: beats per minute); SDNN: standard deviation of all interbeat intervals (ms: milliseconds); RMSSD: the square root of the mean of the sum of the squares of the differences between adjacent intervals; LF: the power of the low-frequency range (ms^2^: millisecond squared); HF: the power of the high-frequency range.

**Table 3:** Participant 2 statistics and contrast for HRV-related parameters.

Parameter	Descriptives		Wilcoxon’s Test		
	Before Intervention	After Intervention	*Z*	*p*	Cohen’s d
HR (bpm)	107.60 ± 2.88	109.50 ± 7.01	−0.405	0.686	0.368
SDNN (ms)	62.10 ± 27.02	40.90 ± 18.42	−1.214	0.225	1.293
RMSSD (ms)	87.94 ± 35.88	50.87 ± 26.15	−1.214	0.225	1.293
LF (ms^2^)	689.6 ± 841.40	308.5 ± 116.47	−0.944	0.345	0.931
HF (ms^2^)	1486.80 ± 2199.47	413.25 ± 272.87	−0.944	0.345	0.931
LF/HF (ms^2^)	1.16 ± 1.53	0.95 ± 0.55	−0.135	0.893	0.121
Stress	9.92 ± 1.18	15.75 ± 7.44	−1.483	0.138	1.772

*p* < 0.05 Cohen’s *d*, considering: small 0–0.99, medium 1–2.49, and large +2.50 [59]. HR: heart rate (bpm: beats per minute); SDNN: standard deviation of all interbeat intervals (ms: milliseconds); RMSSD: the square root of the mean of the sum of the squares of the differences between adjacent intervals; LF: the power of the low-frequency range (ms^2^: millisecond squared); HF: the power of the high-frequency range.

## Data Availability

The datasets used during the current study are available from the corresponding author on reasonable request.
